# Editorial: Pulsed electric field based technologies for oncology applications

**DOI:** 10.3389/fonc.2023.1183900

**Published:** 2023-03-22

**Authors:** Siqi Guo, Gregor Sersa, Richard Heller

**Affiliations:** ^1^ Frank Reidy Research Center for Bioelectrics, Old Dominion University, Norfolk, VA, United States; ^2^ Department of Experimental Oncology, Institute of Oncology Ljubljana, Ljubljana, Slovenia; ^3^ Faculty of Health Sciences, University of Ljubljana, Ljubljana, Slovenia; ^4^ Department of Medical Engineering, University of South Florida, Tampa, FL, United States

**Keywords:** pulsed electric fields, electrochemotherapy, gene electro-transfer, irreversible electroporation, tumor ablation, immune response

Though various pulsed electric field (PEF)-based technologies can be used to treat cancer, there are two distinct sources of therapeutic effects: direct biological actions by PEFs or the cytotoxicity of drugs/production of genes delivered by PEFs. For PEF technologies, such as electrochemotherapy (ECT), gene electrotransfer (GET), and calcium electroporation, the efficiency of tumor ablation depends on the efficiency of the PEF-assisted delivery of chemotherapeutics (bleomycin or cisplatin), therapeutic genes, and calcium into the tumor. On the other hand, for PEF technologies, such as tumor-treating fields (TTFs), irreversible electroporation (IRE), and nanosecond pulsed electric fields or nanopulse stimulation (NPS), the efficiency of tumor ablation depends on the properties of the PEF itself. There may be an argument regarding the moderate or partial contribution of the direct PEF effect in ECT, GET, and calcium electroporation; however, there is no question about the tumor regression by TTFs, IRE, and NPS resulting solely from the PEF effect. In both scenarios, the properties of the PEF are essential to determine the biological effects either directly *via* PEF-induced cytotoxicity or *via* the delivery of therapeutic drugs/genes into tumor tissues.

In this special edition, Novickij et al. discussed the significance of the shape of electric pulses for membrane permeabilization and its potential applications. Advances in pulsed power technology can provide a wide range of PEFs with pulse durations ranging from picoseconds to milliseconds, electric field strengths up to several hundred KV/cm, frequencies up to GHz, and varied waveforms. Focusing on the commonly used square waveforms, the authors described the physical features, biological effects, potential mechanisms, advantages, and engineering challenges of several square waveforms including the unipolar square wave, bipolar square wave, CANCAN (cancellation of bipolar cancellation), and asymmetric pulses. Besides the pulse waveform determining the pulse duration and electric field amplitude, the authors also discussed the effects of frequency or the pulse repetition rate, which are more influential in terms of shorter pulses (<100 µs). In general, cell membrane permeabilization is the foundation of current PEF-based cancer therapies such as ECT, GET, and IRE. On the contrary, ultrashort (nanosecond and picosecond) PEFs can affect intracellular structures/organelles but leave the cell membrane unchanged (1). Another unique feature of ultrashort (picosecond) PEFs is the feasibility of non-invasive delivery by antenna. NPS has been demonstrated to effectively treat cancers in numerous animal models (2–6) and clinical trials (7, Ross et al.). Currently, there are engineering challenges for picosecond PEFs to treat cancer. Nevertheless, we believe these engineering obstacles will be resolved eventually, and this non-invasive ultrashort PEF technology will have broad medical applications, including in cancer therapy (8) and deep brain stimulation (9).

ECT is the most broadly used PEF-based cancer therapy that is approved in many countries, in contrast to other PEF-based therapies such as IRE, GET, calcium electroporation, and NPS, which are currently at various phases in clinical trials. This is also reflected in this special edition. There are four research reports studying the medical applications of ECT. By analyzing the data in the InspECT (International Network for Sharing Practice on ECT) registry, Bertino et al. reported the application of ECT to treat 162 cases of cutaneous squamous cell carcinoma (cSCC). Noticeably, the majority of patients who underwent one or more than one prior therapy still obtained an overall response rate of 83%, with a complete response rate of 62% and a partial response rate of 21%. Further analysis demonstrated that intravenous drug administration is significantly better than intratumoral administration. Tumors with small sizes and ECT as their primary therapy also have better clinical outcomes. As the authors pointed out that this study is not a clinical trial but the analysis of real-world heterogeneous patients with ECT adopted to treat cSCC, some conclusions, such as whether intravenous drug administration is better than intratumoral administration—which appears to contradict with other studies—should therefore be further investigated in well-designed clinical trials.

The most common cancers treated with ECT are surface malignancies (10) including cSCC, basal cell carcinoma, Kaposi’s sarcoma, melanoma, and skin metastatic tumors. Due to the high effectiveness of local tumor clearance and favorable safety features in these superficial tumors, advanced or enhanced ECT technologies have been developed to treat tumors located in deep tissues/organs. Trotovsek et al. reported the use of laparoscopic ECT to treat two cases of unresectable hepatocellular carcinoma (HCC), which are unsuitable with cutaneous ECT or other ablation methods. With advances in electrode designs and their accommodation into laparoscopy, endoscopy, and other minimal invasive diagnostic/therapeutic systems (11), we can envision that ECT will be a therapeutic choice for many other solid tumors regardless of histological types and physical locations. The research article titled “*Combining super selective catheterization and electrochemotherapy: A new technological approach to the treatment of high-flow head and neck vascular malformations*” is an example showing that a technological advance can expand the ECT application to some difficult clinical situations—in this case, high-flow vascular malformations with comorbidities (Krt et al.). Though these two reports are not standard clinical trials, they demonstrated that a well-trained team could take advantage of ECT to manage complex needs for cancer treatment or tissue ablation, while other available therapies face a great challenge. In terms of the clinical management of ECT for cancer therapy, anesthesia is part of the management plan. In a prospective clinical study, Benedik et al. assessed the tolerability, safety, and potential advantages of two anesthetic techniques: general anesthesia and continuously intravenous sedation. They found that both anesthetic methods achieved similar outcomes in terms of the numeric pain score, subjective satisfaction, and adverse effects. Continuous intravenous sedation showed some benefits, including reduced anesthetic quantity (propofol) and a faster recovery; it is particularly helpful for patients with comorbidity who might deteriorate with long-duration anesthesia.

GET is currently in phase II trials for melanoma and triple-negative breast cancer (clinicaltrials.gov) after initially encouraging results from the phase I human trial (12), which indicated that GET is a safe and effective PEF technology for clinical application. Hollevoet et al. reported a large animal (sheep) study on the dosing and pharmacokinetics of GET with the DNA coding antibody. Their study proves that the level of targeted proteins can be tuned by the DNA dose, as previously reported by Dr. Heller’s group (13, 14). It also indicates that a long-term (> one year) protein expression reaching a therapeutic level can be achieved by intramuscular GET if no antidrug–antibody (ADA) response is induced. In this sheep study, the ADA response was observed in only 1 out of 18 or 5.6% of animals, which is similar to (15) or lower than (16) some reported ADA rates for antibody protein therapy in human trials. Antibody therapy has been widely utilized to treat many types of diseases including cancer, infectious diseases, and chronic inflammatory disorders. Major limitations include the high cost and repetitive administrations. One-time GET can generate and maintain a long-term therapeutic level of antibody, which will not only significantly reduce the cost and will avoid repetitive protein administrations. Therefore, GET with the DNA coding therapeutic antibody has great potential in cancer therapy and chronic diseases where the antibody is one therapeutic choice.

We have to emphasize that research in PEF-based technologies and their oncological applications is still emerging. Substantial animal studies and clinical trials suggest that PEF technologies such as ECT, IRE, GET, and NPS can be highly effective at treating local tumors in a minimally invasive manner with few and manageable side effects. Due to the high efficiency and favorable safety features, these PEF-based therapies are advantageous for treating patients who cannot tolerate the current standards of care, such as chemotherapy, radical surgery, and radiotherapy. One trend for the applications of PEF-based technologies is to improve clinical outcomes by developing effective combination therapies with other cancer treatments. Furthermore, more and more data are showing that PEF therapies in certain conditions/models may not only result in local tumor regression but also induce systemic immune responses, which have a distant effect (abscopal tumor regression) and long-term memory (prevention against recurrence). Three decades have passed since the first clinical trial of ECT was approved to treat tumors (17). There have been many breakthroughs and much progress in terms of PEF-based technologies and applications, yet this research field is still wide open, and there are still many questions and challenges. We encourage more oncologists and basic and translational scientists to explore the uses of these technologies and to become dedicated to these promising technologies for better and more effective cancer control.

## Author contributions

All authors listed have made a substantial, direct, and intellectual contribution to the work and approved it for publication.
